# Inguinal Hernia Repair Using Absorbable Biosynthetic Mesh Versus Permanent Polypropylene Mesh: A Preliminary Comparative Study of 1-Year Outcomes

**DOI:** 10.3389/jaws.2025.14836

**Published:** 2025-07-09

**Authors:** Ferdinand Köckerling, Kasia Bradbury, Catherine C. Steele, Daniela Adolf, Amit Badhwar

**Affiliations:** ^1^ Department of Surgery and Center for Minimally Invasive Surgery, Academic Teaching Hospital of Charité Medical School, Vivantes Humboldt-Hospital, Berlin, Germany; ^2^ Becton, Dickinson and Company, Warwick, RI, United States; ^3^ StatConsult, Society for Clinical and Health Services Research mbH, Magdeburg, Germany

**Keywords:** inguinal hernia repair, absorbable mesh, synthetic mesh, hernia, inguinal hernia

## Abstract

**Introduction:**

Inguinal hernia repair with synthetic mesh became a gold standard due to durability and low recurrence rates. With the growing popularity of absorbable meshes in abdominal wall repairs, we evaluated performance of absorbable biosynthetic mesh and permanent synthetic mesh in inguinal hernia repair.

**Methods:**

A retrospective analysis of patients undergoing elective unilateral inguinal hernia operations with absorbable biosynthetic (Phasix™ Mesh and Phasix™ ST Mesh) and permanent synthetic (Bard™ Soft Mesh and Ventralight™ ST Mesh) from Herniamed was performed. Patients in both mesh groups were matched using 1:1 propensity score matching. Complications (intraoperative, general, and postoperative) and clinical outcomes at 1-year follow-up were reported and compared between matched pairs.

**Results:**

Among 1,185,067 patients from 954 centers, 8,286 patients fit the inclusion criteria, 75 patients with absorbable mesh – Phasix group, and 8,211 patients with synthetic mesh – PP group. After propensity score matching, there were 64 matched pairs. There were no statistical differences between groups observed in intraoperative, general, or postoperative complications. At 1-year follow-up, there were no recurrences reported for either group. There were no significant differences in 1-year outcomes including pain (on exertion, at rest, and requiring treatment) between Phasix and PP groups.

**Conclusion:**

Complications and 1-year outcomes following inguinal hernia repair are not statistically different between absorbable biosynthetic and permanent synthetic mesh. Despite the absorbable nature of the biosynthetic mesh, the 1-year outcomes are similar to permanent synthetic mesh. However, the study was conducted on a small cohort and future studies with longer follow-up are needed to evaluate advantages, if any, over the other mesh types.

## Introduction

Inguinal hernia repair is the most common abdominal wall hernia repair in the United States and globally [1–3]. A lifetime risk of developing inguinal hernia is estimated to be 27% for men and 3% for women [3]. The risk factors include old age, both low and high BMI, chronic obstructive airway disease, a positive family history of inguinal hernia, and a higher total activity index [3–5].

Treatment options for inguinal hernia include a watch-and-wait approach in case of minimally symptomatic hernias, to surgical treatments when symptoms intensify and decrease quality of life. When surgical treatment is required, mesh repair is generally preferred over non-mesh repair to reduce the likelihood of the hernia recurring [1, 6, 7]. International Guidelines for Groin Hernia Management recommend mesh use, either by open or laparoscopic technique, in inguinal hernia repair [1].

There is a large variety of mesh products available on the market including synthetic, composite, and absorbable (biologic and biosynthetic) mesh. Synthetic meshes are considered a standard of care in hernia repairs because of their elasticity, tensile strength, relatively low costs, and long-lasting support [1]. Synthetic meshes are mostly made of polymers like polypropylene, polytetrafluoroethylene (PTFE), and polyester. However, inguinal hernia repair with mesh has a potential for mesh-related complications like chronic pain, seroma, infection, or foreign body reaction [2, 8–11].

Absorbable meshes were created to reduce some of the potential negative outcomes caused by permanent, synthetic mesh. Made of biodegradable polymers, biosynthetic meshes are designed to break down overtime. Depending on the type of the polymer, the time of degradation differs: 1–3 months (e.g., polyglactin, polyglycolic acid), 6 months (e.g., polyglycolic acid/trimethylene carbonate), 12–18 months [e.g., poly-4-hydroxybutyrate (P4HB), polyglycolide/polylactide/trimethylene carbonate] [12]. Despite multiple studies reporting similar recurrence rates between permanent and absorbable or partially absorbable meshes, the possibility of hernia recurrence due to lack of tissue support after the mesh degradation is still a concern [13–15].

This study investigates the patients’ outcomes after inguinal hernia repair with absorbable biosynthetic and permanent synthetic mesh to understand if the mesh material is associated with the outcomes of the repair at 1-year follow-up. The absorbable mesh used in this study is made out of poly-4-hydroxybutyrate (P4HB) designed to degrade through hydrolysis within 12–18 months giving tissue the time to constructively and functionally remodel. The permanent synthetic mesh used in this study is a large pore knitted polypropylene mesh, the most commonly used material for hernia repairs, designed to remain in the body indefinitely. Both mesh types come with or without an absorbable hydrogel barrier, based on Sepra^®^ Technology (ST), on the anterior side. This barrier reabsorbs within 30 days and is designed to minimize tissue attachment during this time.

## Methods

### Data Collection

A retrospective analysis of data collected from 1,185,067 hernia surgeries from 954 centers was performed through the Herniamed Quality Assurance Registry.

Herniamed is a multicenter, internet-based hernia registry into which participating hospitals and surgeons engaged in private practice in Germany, Austria, and Switzerland have entered data prospectively on their patients who had undergone routine hernia repair and signed an informed consent agreeing to participate. Documentation and data entry are effected in pseudonymized manner in compliance with the legally binding provisions of data protection so that in no case can conclusions be drawn from the data about the actual patient. As part of the information provided to patients regarding participation in the Herniamed Registry and signing the informed consent declaration, all patients were informed that the treating hospital or medical practice would like to be informed about any problems occurring after the operation and that the patient has the opportunity to attend for clinical examination. Further information on the methods has been described previously [16].

**FIGURE 1:**
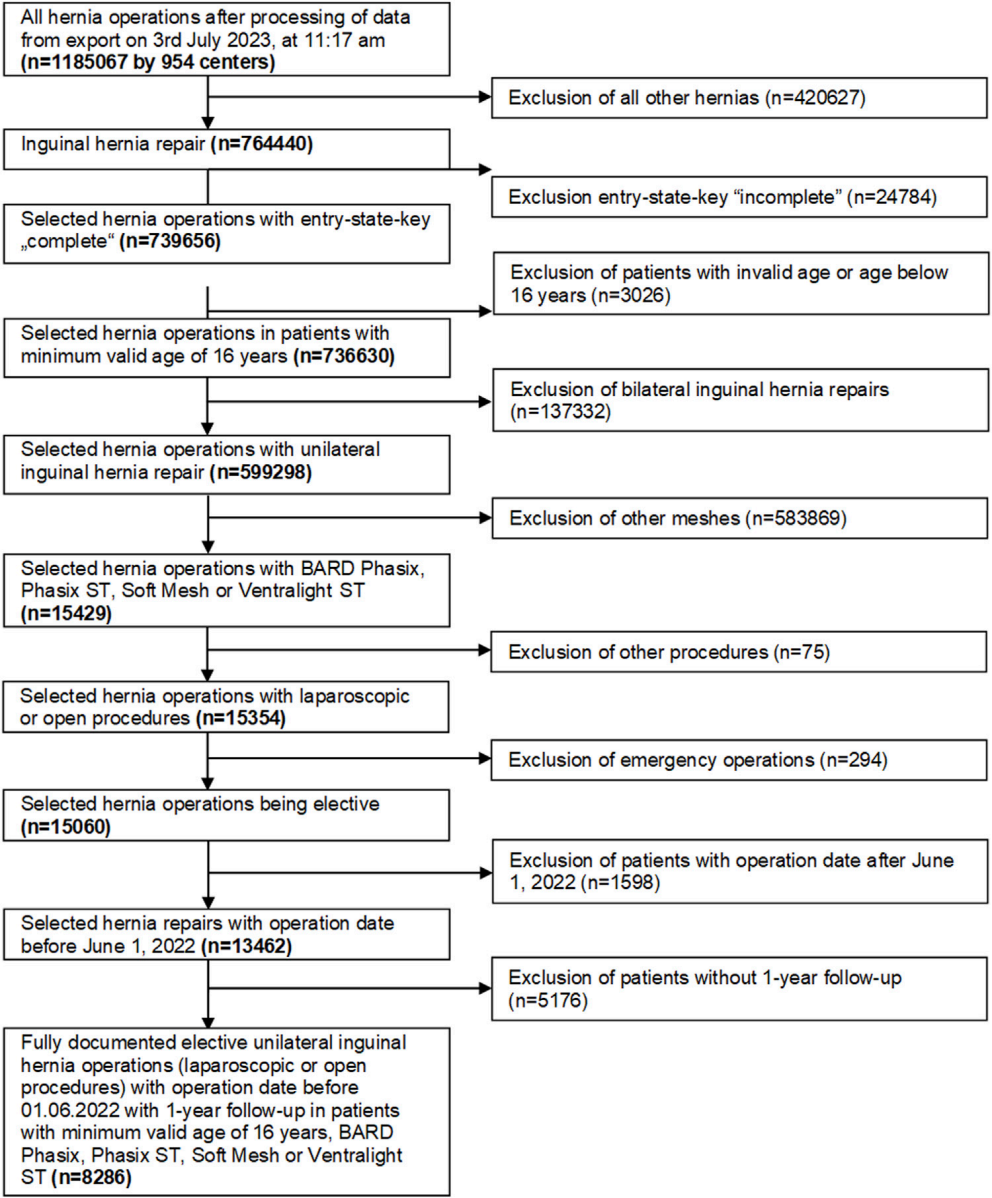
Flow chart of the patient inclusion criteria.

From 764,440 inguinal hernia repair recorded from 5th January 2009 to 6th January 2022, 8,286 procedures fit the inclusion criteria for this analysis (Figure 1). The inclusion criteria included: patients with minimum valid age of 16 years who underwent elective unilateral inguinal laparoscopic or open hernia repair. Patients had hernia repair performed with the use of absorbable biosynthetic mesh (Phasix™ or Phasix™ ST, Becton, Dickinson, and Company, Warwick, RI) or permanent synthetic mesh (Bard™ Soft Mesh or Ventralight™ ST, Becton, Dickinson, and Company, Warwick, RI). The surgeries had to be fully documented, and patients had to have 1 year follow-up.

Operative complications included intraoperative complications, general complications, postoperative complications, and complication-related reoperations. At 1 year follow-up general practitioners and patients completed a questionnaire for outcomes including recurrence, pain on exertion, pain at rest, pain requiring treatment, trocar hernia, secondary hemorrhage, seroma, and infection.

Patients included in the data analysis were divided into two cohorts. The absorbable biosynthetic mesh group included patients who had hernia repair with poly-4-hydroxybutyrate meshes (Phasix™ Mesh or Phasix™ ST Mesh, Becton, Dickinson, and Company, Warwick, RI – collectively “Phasix”), and permanent synthetic mesh group included patients who had hernia repair with lightweight or medium weight monofilament polypropylene mesh (Bard™ Soft Mesh or Ventralight™ ST, Becton, Dickinson, and Company, Warwick, RI – collectively “PP”).

### Statistical Analysis

All analyses were performed with the software SAS 9.4 (SAS Institute Inc., Cary, NC, United States) and intentionally calculated to a full significance level of 5%, i.e., they were not corrected in respect of multiple tests.

For unadjusted homogeneity tests of mesh groups, the chi-square test was used for categorical variables and the robust t-test (Satterthwaite) for continuous variables. Analyses of non-normal distributed data (duration of operation and mesh size) were conducted on log-transformed values.

Pairs of Phasix™/Phasix™ ST and Soft Mesh/Ventralight™ ST were matched in a 1:1 propensity score matching using the robust Greedy algorithm and a caliper of 0.2 standard deviations of the logit of the propensity score. The variables used for matching were as follows: age, American Society of Anesthesiologist classification (ASA), defect size, body mass index (BMI), preoperative pain, risk factors as well as fixed variables: group assignment, sex, type of access and recurrent operation, in which no variability was allowed between matched pairs.

After the propensity score matching the relationship of the mesh groups to the outcome variables was examined. For the categorical outcome variables, the McNemar test (test for systematic deviation between paired patients) was performed to assess whether the mesh groups deviate significantly for an outcome variable.

## Results

A total of 8,286 patients were evaluated (Figure 1). For analysis, patients were divided into 2 groups: Phasix (n = 75 patients treated with Phasix™ or Phasix™ ST) and PP group (n = 8,211 patients treated with Bard™ Soft Mesh or Ventralight™ ST). Propensity score matching was performed to yield relatively homogenous comparison groups. Matching could be implemented for 64 patients for each group representing 85.3% of patients treated with Phasix™ or Phasix™ ST and 0.8% of the comparison group, i.e., patients treated with Bard™ Soft Mesh or Ventralight™ ST. Repairs with Phasix™ mesh (n = 61) were matched only to repairs with Bard™ Soft mesh (n = 61), and repairs with Phasix™ ST (n = 3) were matched only to Ventralight™ ST (n = 3). For the majority of the matching variables, the standardized differences were below 10% (<0.1), which indicates that the variables included in the model are well-balanced between comparison groups.

### Patients Characteristic

Demographic data of matched pairs presented in Table 1 show that patients were primarily male 79.7% in both the Phasix and PP group, with similar age (Phasix: 41.5 ± 17.6 vs. PP: 42.0 ± 16.8, mean standardized difference 5.6 years). BMI ranged from normal to obese/morbid with most of the patients having normal BMI (Phasix: 78.1% vs. PP: 81.3%). Overweight patients were similar in both groups (Phasix: 17.2% vs. PP 18.8%), while there were more obesity/morbid patients in Phasix group than in PP group (4.7% vs. 0.0%, respectively).

**TABLE 1:** Patients’ characteristics and operative details (matching variables) after propensity score matching (n = 64 matched pairs).

	Phasix mesh	PP mesh
	n (%)	n (%)
Phasix^TM^/Bard^TM^ Soft Mesh	61 (95.3%)	61 (95.3%)
Phasix^TM^-ST/Ventralight^TM^-ST	3 (4.7%)	3 (4.7%)
Age [years ± SD]	41.5 ± 17.6	42.0 ± 16.8
Male	51 (79.7%)	51 (79.7%)
BMI		
Normal weight	50 (78.1%)	52 (81.3%)
Overweight	11 (17.2%)	12 (18.8%)
Obesity/Morbid	3 (4.7%)	0 (0%)
ASA score		
I	38 (59.4%)	40 (62.5%)
II	25 (39.1%)	23 (35.9%)
III-IV	1 (1.6%)	1 (1.6%)
Defect size		
I (<1.5 cm)	28 (43.8%)	26 (40.6%)
II (1.5–3 cm)	35 (54.7%)	37 (57.8%)
III (>3 cm)	1 (1.6%)	1 (1.6%)
Type of access		
Laparoscopic surgery	29 (45.3%)	29 (45.3%)
Open surgery	35 (54.7%)	35 (54.7%)
Preoperative pain	47 (73.4%)	43 (67.2%)
No preoperative pain	14 (21.9%)	17 (26.6%)
Unknown preoperative pain	3 (4.7%)	4 (6.3%)
Recurrent operation	7 (10.9%)	7 (10.9%)
Risk factors	11 (17.2%)	12 (18.8%)

Abbreviations: SD, standard deviation; BMI, body mass index; ASA, american society of anesthesiologists; cm, centimeters.

The Phasix group included 59.4% ASA I, 39.1% ASA II, and 1.6% ASA III-IV patients, while the PP group included 62.5% ASA I, 35.9% ASA II, and 1.6% ASA III-IV patients. There was a similar number of patients with risk factors in both groups (Phasix: 17.2% vs. PP: 18.8%). Preoperative pain was reported in the majority of patients (Phasix: 73.4% vs. PP: 67.2%).

### Operative Details

Table 1 also shows the operative details after matching. Patients in both groups had similar defect size. The majority of patients had a defect size classified as II (1.5–3 cm) (Phasix: 54.7% vs. PP: 57.8%). There were 43.8% patients in the Phasix group and 40.6% in the PP group with Defect size I (<1.5 cm). Only 1.6% of patients in both groups were classified as a defect size III (>3 cm).

The majority of patients (57.4%) underwent open hernia repair, while 45.3% patients underwent laparoscopic repair in both groups.

### Operative Complications

Reported complications are presented in Table 2. There were no statistical differences between groups in the rates of complications. There were no intraoperative complications reported in any of the groups. There was a report of general complications in one patient in the PP group where the matched patient had no general complication, and one patient in each group of the matched pairs with postoperative complications where the matched patient had no such complication. There was one patient in the PP group only who required complications-related reoperation, and none in the Phasix group.

**TABLE 2:** Reported complications and 1-year follow-up outcomes (n = 64 matched pairs).

	Concordant cases	Discordant cases		p-value
	Cases in both mesh groups of the matched pair	Cases in phasix mesh only	Cases in PP mesh only	
	n (%)	n (%)	n (%)	
Intraoperative complications	0 (0%)	0 (0%)	0 (0%)	-
General complications	0 (0%)	0 (0%)	1 (1.56%)	0.500
Postoperative complications	0 (0%)	1 (1.56%)	1 (1.56%)	1.000
Complication-related reoperations	0 (0%)	0 (0%)	1 (1.56%)	0.500
Recurrence on 1-year follow-up	0 (0%)	0 (0%)	0 (0%)	-
Pain on exertion on 1-year follow-up	1 (1.56%)	8 (12.5%)	5 (7.81%)	0.581
Pain at rest on 1-year follow-up	0 (0%)	4 (6.25%)	2 (3.13%)	0.688
Pain requiring treatment on 1-year follow-up	0 (0%)	2 (3.13%)	1 (1.56%)	1.000
Trocar hernia on 1-year follow-up	0 (0%)	0 (0%)	0 (0%)	-
Secondary hemorrhage on 1-year follow-up	0 (0%)	0 (0%)	0 (0%)	-
Seroma on 1-year follow-up	0 (0%)	3 (4.69%)	0 (0%)	0.125
Infection on 1-year follow-up	0 (0%)	1 (1.56%)	0 (0%)	0.500

### Outcomes at 1-Year Follow-Up

Outcomes at 1-year follow-up are presented in Table 2. At 1-year follow-up, there were no recurrences reported for both groups.

Other reported outcomes at 1-year follow-up did not differ statistically between the groups, and included discordant cases as follows: pain on exertion on 1-year follow-up (Phasix: 12.5% vs. PP: 7.8% with 1 (1.56%) concordant case where both patients reported yes), pain at rest on 1-year follow-up (Phasix: 6.3% vs. PP: 3.1%), pain requiring treatment on 1-year follow-up (Phasix: 3.1% vs. PP: 1.6%), seroma on 1-year follow-up (Phasix: 4.7% vs. PP: 0.0%), and infection on 1-year follow-up (Phasix: 1.6% vs. PP: 0.0%). There were no reports of trocar hernia and secondary hemorrhage at 1-year follow-up.

No systematic deviation between the comparison groups was demonstrated.

**FIGURE 2:**
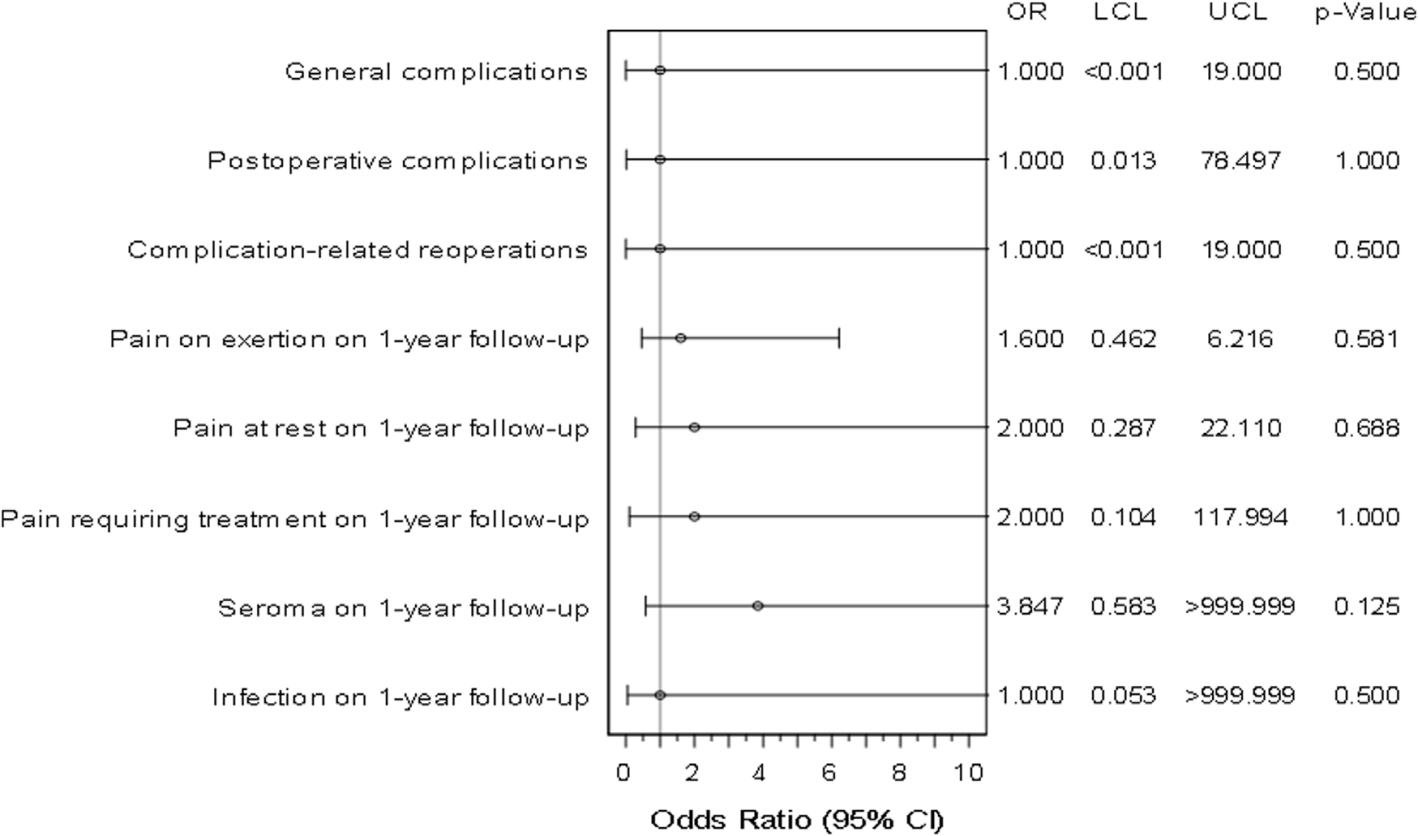
Forest plot - adjusted odds ratios (incl. Confidence interval) for all outcome parameters for Phasix™/Phasix™ ST vs. Soft Mesh/Ventralight™ ST (n = 64 matched pairs). LCL, Lower Confidence Limit. UCL, Upper Confidence Limit.

Odds ratios and confidence intervals were calculated for all outcome parameters. Results are presented in Figure 2. The reported odds ratio values for the Phasix group were not statistically significant and due to the low number of matched pairs, uncertainty presented by wide 95% confidence intervals is relatively high.

## Discussion

Mesh has been used for decades in inguinal hernia repair, with synthetic mesh being the gold standard due to its durability and low recurrence rate when compared to suture repair. Absorbable mesh offers an alternative to permanent synthetic mesh that may reduce chronic pain and foreign body response compared to synthetic mesh, but some are concerned about the durability of repair with an absorbable mesh. Phasix™ Mesh a absorbable biosynthetic mesh has been used in inguinal hernia repair and the authors concluded that Phasix™ is safe and effective for inguinal hernia repair [15]. The current retrospective study compared and analyzed outcomes of inguinal hernia repair between patients with absorbable P4HB (Phasix) mesh and patients with permanent polypropylene mesh at 1-year follow-up. The main finding of this study was that complications and 1-year outcomes are not statistically different between absorbable biosynthetic mesh (P4HB) and permanent synthetic mesh. There were no statistical differences in any outcome measure reported including recurrence, pain or other clinical outcomes including hemorrhage, seroma, or infection.

### Complications and 1-Year Outcomes

There were no significant differences in intraoperative and postoperative complications including recurrence, hemorrhage, seroma, or infection between P4HB and permanent synthetic mesh in this study.

At 1-year follow-up, there were no statistical differences between P4HB and polypropylene mesh in this study for any outcome measure reported including recurrence other clinical outcomes including hemorrhage, seroma or infection. There were no recurrences reported in both matched groups. In the literature, the recurrence rate ranges from 0.5%–15% and it is the highest within the first year after surgery with the reported value of 1.2% [17–20]. With no recurrences reported, the study confirmed the efficiency of inguinal hernia repair with mesh. A previous study on the use of Phasix™ mesh in inguinal hernia repair also reported no recurrences at 2-year follow-up, though the study had a small sample size with only 15 patients [15]. While there is a concern that absorbable mesh may lead to increased recurrence, the current study and meta-analyses comparing absorbable mesh and permanent mesh demonstrate that there is no increased risk of recurrence.

### Chronic Pain

Pain related outcomes reported at 1-year follow-up were not significantly different between matched groups in this study and are consistent with previously reported rates. The incidence of chronic pain after inguinal hernia repair ranges from 6% to 21.71% [8, 21, 22]. In this study the highest incidence had reports of pain on exertion of 12.5% in the Phasix group only compared to 7.8% in the PP group only with an additional case in both patients of the matched pair (+1.56% each), followed by pain at rest with 6.3% in the Phasix group vs. 3.1% in the PP group. The rates of pain requiring treatment on 1-year follow-up were 3.1% in the Phasix group and 1.6% in the PP group.

Chronic pain is not clearly linked to the mesh material, with some reports favoring absorbable meshes in relation to pain while others showing no differences [13, 21]. While there are no randomized controlled trials that favor permanent meshes regarding pain post repair, there are some studies that show pain reduction with absorbable meshes [21]. Different mesh materials could induce different foreign body responses, contributing to the chronic pain experienced by patients [10, 23, 24]. In two meta-analyses, permanent meshes were made out of polypropylene while the absorbable mesh groups were very diverse [13, 21]. Most of the absorbable meshes in both meta-analyses were biological (Surgisis, Acellular extracellular matrix). Other absorbable/partially absorbable meshes included TIGR, Gore Bio-A, Vypro II, and Ultrapro, made of polyglycol, trimethylene carbonate, polyglycolic acid, polyglactin, poliglecaprone. Due to this high variability of absorbable materials, it is difficult to determine whether the results favor a specific type of mesh.

Other studies have reported pain reduction in patients after repair with lightweight mesh compared to heavyweight mesh [8, 9, 13, 25]. A reduced amount of prosthetic material appears to improve pain for patients. Absorbable meshes should have improved results in pain reduction since the implanted material disappears overtime. For example, at 1-year follow-up, Phasix™ mesh is already partially reabsorbed and native tissue strength is regained [26]. Previous studies on Phasix™ Mesh use in laparoscopic inguinal hernia repair showed reduction of reported pain from 8.3% at 1-year to 0% at 30-month follow-up [15]. In the current study, pain in the Phasix group was not significantly different from the PP group in matched pairs at 1 year. Further studies with longer follow-up and larger patients’ groups should be conducted to better understand pain post repair with absorbable and synthetic mesh.

### Limitations

The biggest limitations of this study are retrospective analysis of registry data, the disproportion in numbers of patients in the absorbable and permanent mesh group, and the follow-up duration. Propensity score matching was applied to yield relatively comparable groups for evaluation of outcomes. Since a 1:1 matching was used, the disproportion in the numbers of patients in the groups was addressed, but also in case of a 1:n matching, the relatively small group size of the Phasix group would remain (64 patients), which may lack statistical power to detect significant differences. Further, the matching reduces some of the bias associated with mesh selection criteria, but patient-specific factors not reported in the registry could still affect the product choice and the results.

In addition, longer follow-up is necessary. The current study investigated 1-year outcomes, which is the time period in which most of the absorbable biosynthetic mesh has resorbed. However, longer follow-up is necessary to ensure the results are consistent once the mesh is fully resorbed.

## Conclusion

The findings of this study demonstrate that complications and 1-year outcomes are not statistically different between absorbable biosynthetic mesh (P4HB) and permanent synthetic mesh. However, the study was conducted on a small cohort and future studies with longer follow-up are needed to evaluate advantages, if any, over the other mesh types.

## Data Availability

The original contributions presented in the study are included in the article/supplementary material, further inquiries can be directed to the corresponding author.
